# The impact of mind-body internet and mobile-based interventions on fatigue in adults living with chronic physical conditions: A systematic review and meta-analysis of randomized controlled trials

**DOI:** 10.1371/journal.pdig.0000878

**Published:** 2025-06-11

**Authors:** Serena Isley, Emily Johnson, Shaina Corrick, Ashley Hyde, Ben Vandermeer, Naomi Dolgoy, Nathanael Tabert, Edith Pituskin, Puneeta Tandon

**Affiliations:** 1 Department of Medicine, Division of Gastroenterology (Liver Unit), University of Alberta, Edmonton, Alberta, Canada; 2 Department of Medicine, University of Alberta, Edmonton, Alberta, Canada; 3 Faculty of Rehabilitation Science, University of Alberta, Edmonton, Alberta, Canada; 4 Faculty of Science, Augustana Campus, University of Alberta, Camrose, Alberta, Canada; 5 Faculty of Nursing, University of Alberta, Edmonton, Alberta, Canada; Iran University of Medical Sciences, IRAN, ISLAMIC REPUBLIC OF

## Abstract

Chronic physical conditions (CPCs) are conditions that persist for long periods and may not have a cure. Fatigue is a common symptom experienced by people living with CPCs. Mind-body internet and mobile-based interventions (IMIs) offer an accessible management strategy. The objective of this review was to assess the impact of mind-body IMIs on fatigue symptoms in adults with CPCs. Six databases were searched from inception to July 2024. Inclusion required randomized controlled trials (RCTs) of mind-body IMIs in adults (≥ 18) with CPCs that assessed fatigue pre-and post-intervention using self-report questionnaires. The primary outcome was the standardized mean fatigue change scores (Hedges’ *g*)*.* Sub-group analyses were conducted on CPC type, mind-body technique, fatigue questionnaire, and personnel support level. Meta-regression was performed on IMI length and age. Study quality was assessed using the Cochrane Risk of Bias 2.0 tool. The search retrieved 5239 studies. Seventeen studies met inclusion criteria: 47% neurological (n = 8), 29% cancer (n = 5), and 24% autoimmune (n = 4). Seven studies (41%) included cognitive behavioural therapy (CBT), seven used CBT combined with non-CBT techniques, and three employed non-CBT techniques. Mind-body IMIs led to significant reductions in fatigue (SMD = -0.74 [-1.09, -0.39]; p < 0.0001), with a greater effect in younger participants (p = 0.005). Heterogeneity was moderate to high. In conclusion, mind-body IMIs show promise in reducing fatigue symptoms in adults with CPCs. Further high-quality RCTs, expanding beyond CBT techniques, and using at least one common fatigue scale across conditions, would be helpful in evaluating the impact of IMIs across a broader range of CPCs.

## Author summary

Fatigue is a common and debilitating symptom in individuals living with chronic physical conditions, significantly impacting quality of life. However, management options remain limited. Mind-body wellness techniques such as yoga and cognitive behavioural therapy (CBT), have been explored as potential interventions for fatigue across chronic physical conditions. The internet and mobile-based delivery of these techniques provides a scalable and accessible solution, but their effectiveness as compared to control groups across diverse chronic physical conditions has not been summarized. This systematic review and meta-analysis aimed to address this evidence gap. Among 17 included studies, 14 used CBT either as a stand-alone intervention or combined with other mind-body techniques. Despite moderate to high heterogeneity, mind-body IMIs were associated with significant reductions in fatigue symptoms. Further research is needed to evaluate these promising approaches through a transdiagnostic lens across chronic conditions, and to expand the evidence base for non-CBT techniques.

## 1. Introduction

Chronic physical conditions (CPCs) are complex, long-term health issues that often do not have a cure [1–3]*.* They are a leading cause of morbidity and mortality, responsible for over 70% of deaths worldwide [4,5], with symptoms increasing a patients’ socioeconomic burden [6,7] and reducing their quality of life [8,9]. Fatigue is one of the most debilitating symptoms for individuals living with CPCs, impacting physical, emotional and psychosocial domains [1,2,10]. Fatigue in this context is characterized by a profound and unrelenting exhaustion that worsens over time and is not improved by rest [2]. Systematic reviews have shown that fatigue affects up to 40% of individuals living with cancer [5], liver disease [11], heart disease [12] and other conditions [13]. The management of fatigue remains challenging, with increasing recognition that many causes of fatigue are similar across conditions [10,13,14]. This has led to interest in fatigue management approaches that can be implemented across a range of CPCs, herein referred to as *transdiagnostic interventions* [15].

Mind-body interventions [15] are a potential transdiagnostic, non-pharmacological intervention for fatigue management. Defined by the National Center for Complementary and Alternative Medicine as “a multimodal approach that focuses on the interactions among the brain, mind, body and behaviour” [16], common mind-body techniques include meditation, mindfulness, Tai-chi [17,18], yoga, and cognitive-behavioural therapy (CBT) [16]. Since the COVID-19 pandemic [19], there has been a notable increase in the delivery of mind-body interventions through internet and mobile-based methods [20], with evidence of cost-effectiveness [21]. Online delivery appeals to individuals who may not have access to in-person classes, prefer the convenience of practice at home, or face a significant symptom burden [22,23]. Existing systematic reviews and meta-analyses in the area have contributed greatly to advancing our understanding of fatigue and mind-body interventions in CPCs [17,24–37]. In a review by Bailey and Morris (2024) [35], a large effect size of -1.40 was found for colorectal cancer patients using mind-body therapies during active chemotherapy treatment. Similarly, two other recent reviews reported medium to large effect sizes for the use of tai chi and other mind-body exercises in managing fatigue in chronic fatigue syndrome [36,37] and post-COVID [36]. Due to the inclusion of single CPCs [24–28], evaluations involving non-clinical populations [29,30], a mix of in-person and online delivery methods [17,31], or non-randomized trial designs [32–34], there remains a gap in the literature regarding the effectiveness of internet and mobile mind-body interventions for fatigue compared to control conditions across a range of CPCs.

Accordingly, the primary aim of this systematic review was to evaluate the impact of select mind-body internet and mobile-based interventions (IMIs) on fatigue symptoms across a range of CPCs compared to control conditions. As a secondary aim, subgroup analyses assessed the impact of CPC type, mind-body technique, level of personnel support (i.e., self-guided versus personnel supported), and fatigue scale used. Meta-regressions assessed the impact of age and intervention length. Data on adherence and study characteristics were collected and summarised when available.

## 2. Methods

### 2.1 Search strategy

Six databases (MEDLINE, Scopus, EMBASE, PsycINFO, CINAHL and Cochrane Central Register of Controlled Trials (CENTRAL)) were searched from database inception to October 2022, and updated in in July 2024. No limitations were set on the time frame of published studies searched for or included. The authors developed inclusion and exclusion criteria with guidance from a medical librarian (LD). A comprehensive search was conducted using medical and lay terms for fatigue (e.g., exhaustion, asthenia), keywords related to online/mobile interventions (e.g., internet, app) and various terms for mind-body (see full search strategy in S1 Appendix). Additionally, reference lists of relevant review articles were manually searched for further studies.

### 2.2 Study selection

Articles retrieved during searches were evaluated based on inclusion/exclusion criteria outlined using PICOD [38]. *Population* – Inclusion required adult (≥ 18) participants living with a CPC. CPCs were defined as any long-term health condition that required healthcare intervention. Individuals with chronic mental health conditions (e.g., substance use disorder, schizophrenia) and caregivers and healthcare workers of patients with chronic physical condition(s) were excluded to focus the analysis on those living with CPCs. Chronic fatigue syndrome (CFS) was also excluded, as fatigue is considered the primary etiology of chronic fatigue syndrome rather than a symptom secondary to another condition [39]*. Intervention* – Recognizing the vast range of mind-body techniques, the review was limited to one of the three categories – CBT (CBT or CBT derivatives), “non-CBT” (select non-CBT mind-body techniques including mindfulness-based stress reduction, breathwork, meditation, yoga, Tai-chi, Qigong), and CBT + (combined intervention with techniques from both CBT and non-CBT categories). Interventions had to be delivered through the internet or mobile platforms, excluding digital video discs (DVD) and interventions delivered entirely via teleconference methods. They could be either “self-guided”, which required no personnel support or “personnel-facilitated”. Personnel-facilitated interventions could involve varying levels of support (e.g., weekly phone calls, text reminders, monthly videoconference) from an individual of varying expertise/specialization (e.g., graduate student, therapist); program orientations and technical support were not considered as personnel support. Interventions of any duration were eligible for inclusion, provided they involved more than a single session. *Comparator* – As the purpose was to compare the standalone effectiveness of mind-body IMIs interventions for fatigue, treatment as usual, care as usual or another non-active group (i.e., some educational material) were considered eligible control groups. Trials that evaluated two active interventions (e.g., IMI versus in-person delivery) were excluded. *Outcomes* – Change in fatigue scores from baseline to post-intervention assessed by fatigue-specific questionnaires were required for inclusion. Given the large number of fatigue questionnaires available, all were considered eligible to maximize inclusivity. *Design* – Only RCTs were eligible for inclusion, as these are considered the gold standard for evaluating intervention effectiveness and minimizing bias.

### 2.3 Data extraction

While this is not a Cochrane Review, the recommendations in the Cochrane Handbook for Systematic Reviews of Interventions [38] provided a guiding framework. Two independent reviewers completed the title/abstract and full-text article screening process in a standardized screening form hosted by Covidence [40]. One of the three trained reviewers (SI, EJ, SC) extracted data and a second reviewer verified a random subset of extracted data using a predefined extraction table hosted on Research Electronic Data Capture (REDCap) [41]. Any disagreements during screening and extraction were resolved through discussion with a third author. To ensure consistency and minimize bias, discrepancies and unclear data were clarified with the principal investigator (PT). The primary outcome was the difference in mean scores of fatigue measures with associated 95% confidence intervals (CIs). When reported, demographic characteristics, including those that could impact technology use, such as sex, gender, ethnicity, age, household income, marital status, employment status, level of education, and technology proficiency data were collected.

Intervention characteristics were also collected, including mind-body technique, CPC type, personnel support level, intervention length, study and intervention adherence, and fatigue scale(s). If reported, intention-to-treat data were used. In the case of missing or unclear data, the primary author was contacted to obtain further information. When available, harms data were extracted, including the type and frequency of adverse events.

### 2.4 Risk of bias assessment

Study quality was assessed using the Cochrane Risk of Bias 2.0 tool [38]. Two independent reviewers (SI, SC or EJ) performed these assessments and any discrepancies in risk of bias judgments were resolved through discussion and consensus. The risk of bias was categorized as low, some concerns, or high. In addition to the Cochrane Risk of Bias 2.0 tool, while we did not do any other further formal quality assessments, meta-analyses were performed to assess sources of heterogeneity. Limitations regarding generalizability and sources of clinical or statistical heterogeneity were identified and discussed further in the results.

### 2.5 Data synthesis and statistical analysis

This systematic review and meta-analysis was registered on PROPSERO (CRD42022375590) and follows the Preferred Reporting Items for Systematic Reviews and Meta-Analyses (PRISMA) guidelines (S1 Checklist) [42]. Review manager 5.4 [43] and Stata/BE 17.0 [44] were used for statistical analyses. Data were synthesized using random effects based on Hedges’ *g* statistic, which is used to adjust for differences in scoring methods across outcome primary measures to generate a pooled measure of effect using the standardized mean difference (SMD) model. When data were missing (i.e., change scores) they were calculated in accordance with “Handling Continuous Outcomes in Quantitative Synthesis: Methods Guide for Comparative Effectiveness Reviews” [45,46]. For baseline calculations with no correlation provided, 0.5 was used [45,46]. For RCTs that included multiple follow-up points, the collection point closest to the completion of the intervention was used. When a study used multiple fatigue scales, the first listed fatigue scale was prioritized for the overall meta-analyses. To be inclusive of the multiple fatigue scales included in this review, a meta-analysis was performed to compare fatigue scales that were not necessarily the primary measure and were used >1 time across studies. Heterogeneity was assessed using the statistical chi-squared test and quantified using the I² statistic [47]. Statistical significance was set at 0.05 for hypothesis testing. According to previous recommendations, an effect size of 0.2 was defined as small, 0.5 medium, and 0.8 large [48]. Results from studies grouped according to pre-hoc study-level characteristics were compared using random effects meta-regression (age, intervention length) or stratified meta-analysis sub-grouped by mind-body technique, CPC group, personnel support level, and fatigue scale. These subgroup analyses and meta-regressions were conducted to explore sources of heterogeneity across studies. Age and intervention length were chosen as previous research suggests that age may affect both the uptake [49,50] and adherence [51] to digital interventions, potentially influencing outcomes. Similarly, intervention duration may affect its effectiveness [52].

## 3. Results

### 3.1 Study characteristics

**Fig 1:**
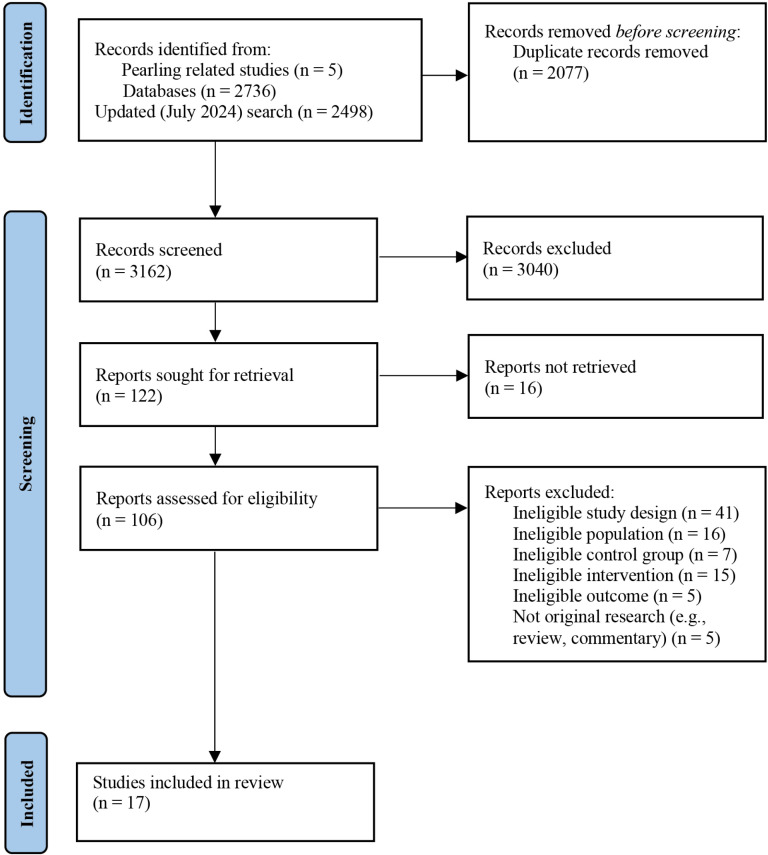
PRISMA flow diagram of the study selection in this review.

Database screening and manual searches yielded 5239 articles. Of these, 3,162 studies were screened at the title and abstract stage, with 106 studies included for full-text review. At full-text, 89 studies were excluded due to i) ineligible study design (i.e., protocols, qualitative, feasibility design) and/or ineligible population, control group, outcome, or intervention type (i.e., intervention delivered entirely via teleconference, entirely personnel supported). Seventeen (n = 17) RCTs met inclusion criteria, involving 1805 participants (Fig 1), and published from 2010 to 2024. Seventy-five percent (n = 1357) were female and 25% (n = 448) were male. Participants ranged in age from 20 to 82 years (mean = 48 years) and lived with the following chronic conditions: cancer (29%) [53–57], multiple sclerosis (23%) [58–61], fibromyalgia (12%) [62,63], epilepsy (12%) [64,65] rheumatoid arthritis (6%) [66], psoriasis (6%) [67], type 1 diabetes (6%) [68], and primary biliary cholangitis (6%). Studies were published from 2010 [63] to 2024 [65] across 7 countries: United States (n = 4) [54,55,61,63], Netherlands (n = 4) [53,66–68], Germany (n = 4) [56,58,60,64], Canada (n = 2) [62,69], the United Kingdom (n = 1) [59], Switzerland (n = 1) [57] and Turkey (n = 1) [65]. Sample sizes ranged from 40 to 275 (mean = 106). Most studies were two-armed RCTs (n = 15, 88%), except one 3-armed RCT [53] and one 4-armed RCT [55]. This review did not include the third and fourth arms as they assessed the intervention effect on a non-clinical population [53,55]. Control participants were either wait-list control (n = 13) or treatment/care as usual (n = 4).

### 3.2. Study interventions

Four of the seventeen studies [54,55,58,64] used mind-body IMIs that were commercially available (e.g., Headspace), while the remainder developed interventions specific to the research study. Most interventions (n = 10) [53,54,57,59,62,65–69] were supported in some capacity by study personnel, with support ranging from monthly face-to-face sessions with a therapist (in conjunction with the IMI) to weekly virtual contact via teleconference, email, text, or personalized feedback on IMI content, (e.g., homework assignments). Seven studies (41%) [55,56,58,60,61,63,64] were entirely self-guided without additional support from research personnel. Seven studies used CBT as the sole intervention [53,59,60,62,66–68]. Seven studies [56–58,61,63,64,69] incorporated additional mind-body techniques to the central CBT intervention, categorized as CBT + . These additional interventions included mindfulness [58,64], yoga [56,69], Tai Chi [69], Qigong [56], meditation [69], relaxation exercises [56,58,63], and mindfulness-based stress reduction [57,61]. Three interventions used only non-CBT techniques [54,55,65]: yoga [54], mindfulness-based stress reduction therapy [55] and progressive muscle relaxation [65]. A minority (n = 5, 29%) of studies assessed fatigue as a primary outcome [57,59,66–68], with 47% (n = 8) [53,56,58,60–64] assessing it as a secondary outcome. Although three studies [54,55,65] did not explicitly list fatigue as a primary or secondary outcome, they included questionnaires that assessed fatigue, allowing for the extraction of relevant data for synthesis. Intervention length ranged from 4-26 weeks (median 12 weeks). See Table 1 for more details.

**Table 1:** Characteristics of included studies.

Author (Year)	Country	Total Sample^a^	Intervention Type	Intervention Duration	Conditions	Personnel Support Level
Boele et al. (2018) [53]	Netherlands	82	CBT (PST- described as a low-intensity CBT)	5 weeks	Cancer (Glioma)	**Personnel-facilitated** **Personnel**: A researcher-psychologist, nurse or trained, supervised psychology students **Facilitation:** Online support and feedback on exercises within 3 working days and additional support upon request of participant
Ferwerda et al. (2017) [66]	Netherlands	133	CBT	26.07 weeks^c^	Autoimmune (Rheumatoid arthritis)	**Personnel-facilitated** **Personnel:** Six female psychologists with a Master’s degree in clinical psychology under the supervision of a senior clinical psychologist with post-academic training in CBT **Facilitation:** Weekly or biweekly email messaging
Fischer et al. (2015) [58]	Germany	90	CBT+ (Deprexis)	9 weeks	Neurological (Multiple Sclerosis)	**Self-guided**
Friesen et al. (2017) [62]	Canada	60	CBT (Pain Course)	8 weeks	Neurological (Fibromyalgia)	**Personnel-facilitated** **Personnel:** A trained and supervised doctorate-level clinical psychology graduate student who was introduced as a guide to participants **Facilitation**: Five to ten-minute telephone contact on a weekly basis
Huberty et al. (2019) [54]	United States	48	Non-CBT (Yoga)	12 weeks	Cancer (Myeloproliferative neoplasm)	**Personnel-facilitated** **Personnel:** Physician specialized in myeloproliferative neoplasms, PhD trained researcher/certified yoga instructor and MS (multiple sclerosis) trained biomechanist/yoga educator **Facilitation**: Develop weekly prescriptions for yoga exercises
İșcan Ayyildiz et al. (2024) [65]	Turkey	60	Non-CBT (PMR)	6 weeks	Neurological (Epilepsy)	**Personnel-facilitated** **Personnel:** Researcher **Facilitation:** Researchers followed up with participants via phone for 6 weeks and participants received notifications (i.e., text messages) to watch the intervention content everyday
Kubo et al. (2019) [55]	United States	97	Non-CBT (Headspace,)	8 weeks	Cancer (multiple types)	**Self-guided** ^b^
Menting et al. (2017) [68]	Netherlands	120	CBT (Dia-fit)	20 weeks	Autoimmune (Type 1 Diabetes)	**Personnel-facilitated** **Personnel:** Three clinical psychologists licensed in CBT **Facilitation**: Five to eight (5.4^*c*^) 50-minute face-to-face sessions with a therapist over 5 months
Moss-Morris et al. (2012) [59]	United Kingdom	40	CBT	10 weeks	Neurological (Multiple Sclerosis)	**Personnel-facilitated** **Personnel:** An assistant psychologist who received 5 hours of basic training in the interventions with fortnightly supervision from a registered psychologist **Facilitation:** Automated emails encouraging the completion of one session a week over 8–10 weeks, as well as 3, 30–60-minute telephone sessions at week 1,3 and 6 of the IMI
Neubert et al. (2023) [56]	Germany	157	CBT+	4 weeks	Cancer (multiple types)	**Self-guided** ^***b***^
Pöttgen et al. (2018) [60]	Germany	275	CBT	12 weeks	Neurological (Multiple Sclerosis)	**Self-guided**
Schröder et al. (2014) [64]	Germany	78	CBT+ (Deprexis)	9 weeks	Neurological (Epilepsy)	**Self-guided**
Titcomb et al. (2023) [61]	United States	100	CBT+	12 weeks	Neurological (Multiple Sclerosis)	**Self-guided**
Urech et al. (2018) [57]	Switzerland	129	CBT+	12 weeks	Cancer (multiple types)	**Personnel-facilitated** **Personnel:** Therapists **Facilitation:** Weekly feedback delivered via email
van Beugen et al. (2016) [67]	Netherlands	131	CBT	25 weeks^*c*^	Autoimmune (Psoriasis)	**Personnel-facilitated** **Personnel:** Therapist (psychologist) **Facilitation:** (1) Two initial face-to-face sessions; (2) Telephone-based introduction to the IMI; (3) Personalized weekly feedback on assignments
Watt 2023 [69]	Canada	87	CBT+	12 weeks	Autoimmune (Primary Biliary Cholangitis)	**Personnel-facilitated:** **Personnel:** Program facilitator **Facilitation:** 10 minute weekly check-ins. Included discussion of weekly content, reviewing progress and facilitating goal-setting
Williams et al. (2010) [63]	United States	118	CBT+	24 weeks	Neurological (Fibromyalgia)	**Self-guided**

^a^Total sample reported in study characteristics.

^b^Only received support for program introduction and/or for technological issues.

^c^Mean intervention duration, no intervention length indicated.

CBT: cognitive behavioral therapy; CBT + : cognitive behavioral therapy + other non-CBT mind-body technique.

PST: Problem-solving therapy; PMR: progressive muscle relaxation.

### 3.3 Fatigue scale characteristics

Thirteen distinct self-report scales or subscales were used to assess fatigue symptoms. Although validation was not a criterion for inclusion, all thirteen of the included scales were validated instruments. Distinctions between these scales, including number of items, score ranges, fatigue recall time frames (e.g., 4 weeks), and dimensions of fatigue (e.g., cognitive, physical), are outlined in Table 2. All surveys required participants to recall the impact and/or experience of fatigue symptoms over a specified time frame, from the past 2 weeks to as long as 4 weeks. All surveys used a Likert scale to quantify fatigue, and total scores varied from 0 to a maximum of 160.

**Table 2:** Fatigue characteristics of included studies.

Author (Year)	Scale(s) used to measure fatigue	Type	Items	Score range	Reference time	Dimension(s) of fatigue
Boele et al. (2018) [53]	CIS (20-item)	7-point Likert	20-item	20-140	Past 2 weeks	Subjective fatigue (8-items), concentration (5-items), motivation (4-items), physical activity (3-items)
Ferwerda et al. (2017) [66]	CIS (8-item)	7-point Likert	8-item	8-56	Past 2 weeks	Subjective fatigue
Fischer et al. (2015) [58]	FSMC; HAQUAMS	5-point Likert; 5-point Likert	20 item; 4-item	20-100; 4-20	No fixed time frame: “In general”; Last 7 days	Cognitive (10-items), motor (10-items); fatigue and cognitive functioning
Friesen et al. (2017) [62]	FSI	11-point Likert	14-item	0-143	Past week	Perceived severity, frequency, and interference with daily functioning
Huberty et al. (2019) [54]	MPN Symptom Assessment	11-point	1-item	0-10	Past 24 hours	Weariness and/or tiredness
İșcan Ayyildiz et al. (2024) [65]	FSS	7-point	9-item	9-63	Within the last week	Impact of fatigue on functioning, and relation of fatigue to motivation, physical activity, social life, family and work
Kubo et al. (2019) [55]	BFI	11-point Likert	9-item	0-10	Past 24 hours	Fatigue level/severity; interference with daily living
Menting et al. (2017) [68]	CIS (8-item)	7-point Likert	8-item	8-56	Past 2 weeks	Subjective fatigue
Moss-Morris et al. (2012) [59]	Chalder Fatigue Scale; MFIS	4-point Likert; 5-point Likert	11-item; 21-item	0-33; 0-84	Present; past 4 weeks	Physical and mental fatigue severity; fatigue impact (cognitive, physical and psychosocial functioning)
Neubert et al. (2023) [56]	EORTC QLQ-FA12	4-point	12-item	0-100	Past week	Physical, cognitive and emotional aspects
Pöttgen et al. (2018) [60]	Chalder; FSMC; HAQUAMS	4-point Likert; 5-point Likert; 5-point Likert	11-item; 20-item; 4-item	0-33; 20-100; 4-20	Present; No fixed time frame: “In general”; Last 7 days	Physical and mental fatigue severity; cognitive (10-items), motor (10-items); fatigue and cognitive functioning
Schröder et al. (2014) [64]	QOLIE-31	7-point Likert	4-item	4-24	Past 4 weeks	Energy/fatigue
Titcomb et al. (2023) [61]	FSS, MFIS	7-point Likert; 5-point Likert	9-item; 21-item	9-63; 0-84	Within the last week; Past 4 weeks	Impact of fatigue on functioning, and relation of fatigue to motivation, physical activity, social life, family and work; assesses the physical, cognitive and psychosocial dimensions of fatigue
Urech et al. (2018) [57]	FACIT-F	5-point Likert	40-item	0-160	Past 7 days	Fatigue in daily activities and function
van Beugen et al. (2016) [67]	CIS (8-item)	7-point Likert	8-item	8-56	Past 2 weeks	Subjective fatigue
Watt et al. (2023) [69]	MFIS	5-point Likert	21-item	0-84	Past 4 weeks	Assesses the physical, cognitive, and psychosocial dimensions of fatigue
Williams et al. (2010) (64)	MFI	5-point Likert	20-item	20-100	“Feeling lately”	General, physical, reduced activity, reduced motivation, mental fatigue

BFI: Brief Fatigue Inventory; CIS: Checklist Individual Strength; EORTC QLQ-FA12: EORTC Quality of Life Questionnaire FA12; FACT-Fatigue: Functional Assessment of Cancer Therapy - Fatigue; FSMC: The Fatigue Scale for Motor and Cognitive functions; FSI: Fatigue Symptom Inventory; FSS: Fatigue Severity Scale; MFI: Multidimensional Fatigue Inventory; MFIS: Modified Fatigue Impact Scale; MPN Symptom Assessment: Myeloproliferative Neoplasm Symptom Assessment; QOLIE-31: The Quality of Life in Epilepsy Inventory; HAQUAMS: Hamburg Quality of Life Questionnaire in Multiple Sclerosis (fatigue and thinking subscale).

### 3.4 Meta-analysis outcomes

#### 3.4.1 *Effects on fatigue.*

**Fig 2:**
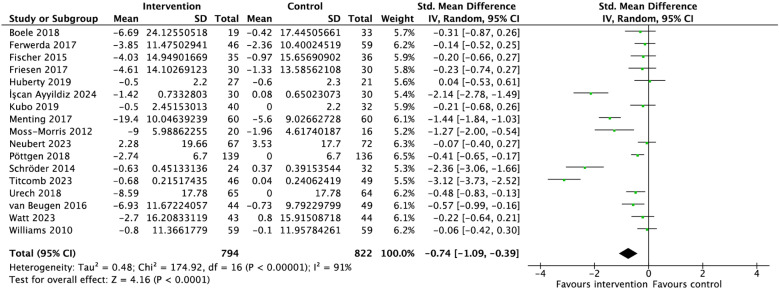
Forest plot of the overall effect of mind-body interventions on fatigue outcomes.

Of seventeen studies, eight showed statistically significant reductions in symptoms of fatigue from baseline to post-intervention (SMD = -0.74 [-1.09, -0.39], p < 0.0001 I² = 91%; Fig 2). All but one study favoured the mind-body IMI over control.

#### 3.4.2 *Subgroup analysis of fatigue measures.*

Six of the thirteen different fatigue scales and/or subscales included in this review were used more than once. Specifically, these six scales appeared in fourteen of the seventeen studies. A subgroup analysis was performed to evaluate the impact of these commonly used fatigue scales (S2 Appendix). The most frequently used scales were the Checklist Individual Strength (CIS) (31%) (8 item scale n = 3 [66–68], 20 item scale n = 1 [53]), Modified Fatigue Impact Scale (MFIS) (n = 3, 23%) [59,61,69], Chalder Fatigue Scale (CFS) (n = 2, 15%) [59,60], the Fatigue Scale for Motor and Cognitive Functions (n = 2, 15%) [58,60], the Fatigue Severity Scale (FSS) (n = 2, 15%) [61,65] as well as the Hamburg Quality of Life Questionnaire in Multiple Sclerosis (HAQUAMS) fatigue and thinking subscale (n = 2, 15%) [58,60] (S2 Appendix). Subgroup differences were observed in this analysis (p = 0.0003, I² = 78.2%). The FSS showed the largest reductions in fatigue (SMD = -2.64 [-3.60, -1.67], p < 0.0001, I² = 79%).

#### 3.4.3 *Subgroup Analysis of mind-body intervention types.*

The use of CBT+ interventions (n = 7) [56–58,61,63,64,69] conferred an advantage over control conditions on fatigue (SMD = -0.89 [-1.60, -0.18], p = 0.010, I² = 95%). Similarly, the use of CBT only interventions (n = 7) [53,59,60,62,66–68] also showed a significant reduction in fatigue compared to control (SMD = -0.60 [-0.96, -0.24], p = 0.0010, I² = 80%). A reduction in fatigue was also evident in the non-CBT interventions [54,55,65] (SMD = -0.75 [-1.99, 0.48], p = 0.23, I² = 93%) but findings were not statistically significant (S3 Appendix).

#### 3.4.4 *Subgroup analysis on levels of personnel support.*

A sub-group analysis assessed the impact on fatigue of interventions that offered personnel support (n = 10) [53,54,57,59,62,65–69] and those that were self-guided (n = 7) [55,56,58,60,61,63,64] (S4 Appendix). No subgroup differences emerged between these groups (p = 0.57; I² = 0%), and the effect on fatigue symptoms remained statistically significant in both subgroups (self-guided SMD = -0.88 [-1.54, -0.21], p = 0.010; personnel-supported SMD = -0.65 [-1.03, -0.27], p = 0.0008).

#### 3.4.5 *Subgroup analysis based on chronic physical condition type.*

The included CPCs were divided into three categories based on frequency: neurological (n = 8) [58–65], autoimmune conditions (n = 4) [66–69], and cancer (n = 5) [53–57] (S5 Appendix). Significant subgroup differences were identified (p = 0.02), but substantial heterogeneity (I² = 74%) was present. Both the cancer and neurological subgroups showed significant reductions in fatigue (cancer SMD = -0.23 [-0.41, -0.04], p = 0.020 and neurological SMD = -1.19 [-1.90, -0.48], p = 0.0010). The autoimmune chronic condition subgroup showed a medium effect size, though it was not statistically significant (SMD = -0.59 [-1.18, -0.00]; p = 0.05).

#### 3.4.6 *Meta-regression of effects on fatigue: intervention length and mean age.*

**Fig 3:**
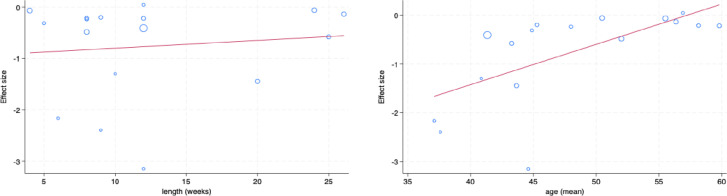
Meta regressions. A. Effect of intervention length on fatigue slope = 0.015, p = 0.67. B. Effect of mean age on fatigue slope = 0.083, p = 0.0050.

The meta-regression results demonstrated a positive but non-statistically significant relationship between intervention length and fatigue (slope = 0.015, p = 0.67) (Fig 3A). A meta-regression on mean participant age identified that younger participants experienced a greater impact on fatigue symptoms following the intervention (slope = 0.083, p = 0.0050) (Fig 3B).

#### 3.4.7 *Adherence data.*

A subgroup analysis was attempted to evaluate the impact of adherence on the intervention. However, the reports and data available were heterogeneous and limited across RCTs. Adherence was collected through self-report, personnel report, and IMI automated tracking. Definitions of adherence across studies were variable, including the average time spent on the IMI, the number of times participants accessed material/content, the number of skills participants used from the online intervention, and completion rates. Due to high clinical heterogeneity across these reports and low comparability, these results could not be statistically analyzed and instead are presented descriptively in S6 Appendix.

#### 3.4.8 *Demographic data.*

Demographic details, including sex, ethnicity, household income, marital status, employment status, level of education, and technology proficiency, were collected (S7 Appendix). Fourteen studies [53–55,57,58,60,62–69] reported education levels through various means (i.e., education in years, percentage of sample with the highest completed level of education). Ten studies reported marital status [54,55,57,59,60,62,63,65,67,69]. A small number of studies reported other sociodemographic variables, those which may impact technology engagement, including employment status (n = 5) [59,60,62,65,69], income (n = 3) [55,57,65] and technology proficiency (n = 4) [59,62,64,66]. Due to variability in report types and the limited data available across these demographic factors, a meaningful statistical comparison could not be conducted.

### 3.5 Risk of bias

**Fig 4:**
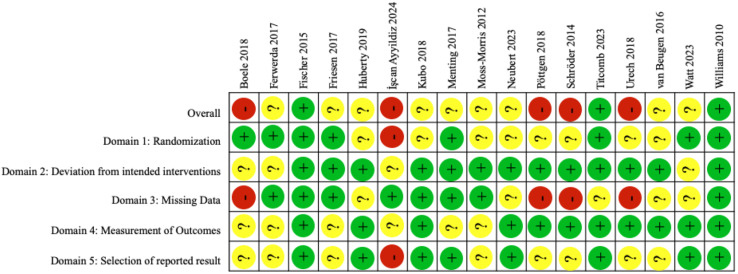
Assessment of risk of bias of included studies. Green circles with ` + ` indicate low risk of bias, yellow circles with `?` indicate some risk of bias and red with `- ` indicates high risk of bias.

The Cochrane Risk of Bias 2.0 tool [38] was used to appraise study quality. Of the seventeen studies, three [58,61,63] had a low risk of bias, nine [54–56,59,62,66–69] had some concerns, and five [53,57,60,64,65] had a high risk of bias (Fig 4). In the fourteen studies with some concerns and high risk of bias, issues arose from randomization (Domain 1) (n = 9 studies), deviation from intended interventions (Domain 2) (n = 4 studies), missing data (Domain 3) (n = 9 studies), measurement of outcomes (Domain 4) (n = 6 studies) and selection of reported result (Domain 5) (n = 9 studies) (Fig 4).

### 3.6 Harms

Of the seventeen studies in this review, 41% (n = 7) [54,57–61,68] discussed adverse events, with adverse events occurring in four of these studies [57,58,60,68] and no adverse events occurring in the remaining [50,55,57]. In the four studies where an adverse event occurred, the types of events and reporting methods varied. Two studies [58,60] used worsening scores on psychometrically validated mental health questionnaires (i.e., worsening Hospital Anxiety and Depression Scale or Beck Depression Inventory scores). The other two studies identified adverse physical health events [54,59]. Adverse event data was collected through various methods, including participant [60,70] and research personnel self-reporting [54,68], and at different time points, including prespecified intervals [68,70], continuously [54], or at the end of study surveys [60].

## 4. Discussion

### 4.1 *Summary of main results*

In this systematic review and meta-analysis of 17 RCTs and 1805 adults living with CPCs, mind-body IMIs were associated with a statistically significant reduction in fatigue symptoms compared to control (SMD = -0.74, p < 0.0001). In the subgroup analyses, fatigue symptoms were reduced across different CPCs, especially in the cancer (SMD = -0.23, p = 0.020). and neurological subgroups (SMD = -1.19, p = 0.0010), and regardless of whether the intervention was self-guided (SMD = -0.88, p = 0.010) or personnel-supported (SMD = -0.65, p = 0.0008). Meta-regression reported a statistically significant reduction in fatigue symptoms in younger adults compared to older adults. While the substantial heterogeneity and small number of studies limit definitive conclusions, the findings support mind-body IMIs as a promising transdiagnostic treatment for fatigue and raise several considerations for discussion.

As a multifaceted symptom with a high burden on patients and limited pharmacological interventions, the management of fatigue in CPCs is challenging. Continued inquiry is required to identify accessible, effective, and broadly applicable therapies. Uniquely, our review focused on mind-body techniques delivered through IMIs, a strategy that can enhance the convenience, flexibility, spread, and scale of health and wellness programming, particularly relevant in individuals with fatigue. Meta-regression revealed a more substantial effect on mitigating fatigue in younger individuals. This association is of interest and aligns with previous research showing younger adults tend to have greater uptake [49,50] and adherence [51] to online interventions. It is plausible that this increased uptake may be influenced by enhanced engagement, digital literacy [71], and technology learning abilities [72] in younger versus older populations. The collection of relevant demographic data that may impact technology use should be a routine part of IMI studies as it can offer valuable insights into generalizability.

This review took a transdiagnostic approach, with all interventions divided into three main CPC subgroups– neurological, cancer, and autoimmune conditions. Recognizing the high prevalence of multi-morbidity in adults with a CPC [73,74] and the shared factors that cause fatigue, this transdiagnostic perspective has become more commonly considered in the recent fatigue literature [75–77]. While the evaluation of mind-body IMIs across diverse chronic conditions may increase heterogeneity, it also has potential to deepen the understanding of which patients may benefit across conditions and lead to an intervention that can be more broadly disseminated across a range of CPCs. Cognitive behavioural therapy and CBT+ interventions predominated in this review, accounting for all but two of the included trials. The current review’s effect size of -0.74 for IMI interventions was higher than the results reported in a 2018 review of systematic reviews of fatigue management (0.24 to 0.48) [15] and similar to the effect size reported in a 2023 systematic review of physical activity (0.54) [78].

The small number of studies in the IMI subgroups (non-CBT, CBT, CBT+) does not allow for firm conclusions about their relative benefit. Experts have suggested that these approaches may be synergistic and have combined merit [79], and various organizations have recommended these therapies as first-line options for managing fatigue [80–82]. With only three non-CBT IMIs included in this review, there is insufficient evidence to clarify their impact on online delivery. Of promise is that non-CBT IMIs do have an impact when delivered in a non-online setting [26,27,83]. For example, a systematic review of 245 studies of exercise and other non-pharmaceutical interventions for cancer-related fatigue in patients during and after cancer treatment identified mind-body relaxation exercises as the most effective intervention that was provided during cancer treatment (SMD = -0.77, range -1.22 to -0.31), and yoga as the most effective following cancer treatment (SMD = -0.68, range -0.93 to -0.43) [84]. Techniques such as tai chi, yoga and other mind-body interventions have shown promise in managing fatigue in specific conditions. While considerable evidence exists on CBT for fatigue [15,80,81], future RCTs should explore a broader range of mind-body techniques to assess fatigue outcomes across various CPCs.

This review included thirteen different fatigue scales. Considering the 252 different fatigue scales identified in the literature [84], this quantity and diversity are unsurprising. While important for understanding symptom experience [85], well-accepted limitations of these scales include their limited generalizability across chronic conditions, and for unidimensional scales, the limited dimensions of fatigue that they address [86,87]. Some scales are more frequently used in specific CPCs, such as using FSS in multiple sclerosis or the BFI in cancer [84]. While there is currently no accepted gold standard to assess fatigue symptoms across a range of CPCs [88], scales such as CIS, FSS and Chalder Fatigue Scale are some of the most commonly used across CPCs [13,84]. Keeping survey burden in mind, future studies evaluating fatigue across multiple CPCs should consider including both a multidimensional and a relevant unidimensional scale. Multidimensional scales, like the Modified Fatigue Impact Scale offer a comprehensive assessment across cognitive, physical, and psychosocial domains and have been applied in diverse chronic condition populations [89,90]. Unidimensional scales with validity across conditions, such as the Fatigue Severity Scale [91–93], can complement this by measuring fatigue severity, frequency, or duration. For single-condition studies, fatigue measurements should be psychometrically validated for the specific target population [94,95]. For example, the 13-item FACIT Fatigue Scale has demonstrated reliability for individuals with cancer. Ultimately, no single tool is sufficient across all chronic populations due to the varied causes, manifestations, and individual experiences of fatigue. A thoughtful approach remains essential for capturing the complexity of fatigue.

### 4.2 Strengths and limitations

This review’s strengths include its focus on RCTs evaluating online mind-body interventions. The findings demonstrated significant effects, with a medium to large effect size (SMD = -0.74). Moreover, the inclusion of eight different CPCs supports a transdiagnostic approach, incorporating both CBT and non-CBT interventions, as well as diverse fatigue scales. While these factors enhance the scope and inclusivity of the review, they also introduce limitations, including surveys and questionnaires that may not all be validated for the populations they were used in and the heterogeneity observed in most analyses. Moreover, there was a high risk of bias or concerns in 14 of the 17 studies. To mitigate bias in mind-body intervention studies, future efforts should focus on including fatigue scales that are validated for the population of interest, using computer-generated sequences for randomization, blinding outcome assessors unaffiliated with the intervention, and implementing comparative effectiveness trials or active control interventions to mask group allocation for participants. Additionally, only 41% of studies reported on adverse events, our ability to draw firm conclusions about these.

### 4.3 Future directions

This review provides a strong foundation for future research, by demonstrating the impact of online mind-body interventions on fatigue in diverse chronic conditions as compared to control. To strengthen the evidence base, future transdiagnostic RCTs should incorporate larger sample sizes, include a multidimensional fatigue scale, prioritize adverse event reporting, and present findings with enhanced clarity and methodological rigor (i.e., blinding, computer generated randomization) to minimize bias. Additional evaluation is also required on non-CBT interventions. As the literature expands, comparing online interventions to in-person alternatives will be of interest to determine the relative effectiveness of these two modalities.

## 5. Conclusions

Fatigue is a debilitating symptom that affects the daily life of people with CPCs and lacks standardized, effective management strategies. Across seventeen studies and 1805 adults living with chronic physical conditions, this systematic review and meta-analysis of the impact of mind-body IMIs on fatigue symptoms reports a medium to large effect size of -0.74. While the high heterogeneity of the studies, the risk of bias, and the use of multiple scales require cautious interpretation, the results of this review offer promise that mind-body IMIs may positively impact this burdensome symptom and deserve further consideration as an accessible and effective management strategy.

## Supporting information

S1 Appendix Full Search Strategy.
(DOCX)

S2 Appendix Subgroup analysis of fatigue measures.
(DOCX)

S3 Appendix Subgroup analysis of mind-body intervention types.
(DOCX)

S4 Appendix Subgroup analysis on levels of personnel support.
(DOCX)

S5 Appendix Subgroup analysis by chronic physical condition type.
(DOCX)

S6 Appendix Adherence data.
(DOCX)

S7 Appendix Demographic data.
(DOCX)

S1 Checklist Prisma Checklist.
(DOCX)
