# Supplementary material for: The impact of mind-body internet and mobile-based interventions on fatigue in adults living with chronic physical conditions: A systematic review and meta-analysis of randomized controlled trials
Source: PLOS Digit Health. 2025 Jun 11;4(6):e0000878. doi: 10.1371/journal.pdig.0000878 (PMC12157242; doi:10.1371/journal.pdig.0000878)
Supplement: S1 Appendix — Full Search Strategy. (DOCX) [file pdig.0000878.s001.docx]

| **Medline search strategy** |
| --- |
| 1. Exp mind-body therapies/ or exp breathing exercises/ or exp meditation/ or ("meditat*" or "wellness intervention" or "wellness therapy" or "wellness program" or mindful* or relaxation or "relaxation therapy" or yoga or "tai ji" or qigong or "breath* exercises" or mind-body or body-mind or "tai chi" or taichi or qigong or breath* or "guided imagery" or "acceptance commitment therapy" or "cognitive behavioural therapy" or "cognitive behavioral therapy" or vitalism or "meditative practice" or samu or "positive psycholog*" or "intentionally focused awareness" or simonton or visuali?ation or imagery or sophrology or "muscle contract*" or "hold relax" or "alternate nostril" or "abdominal breath" or neidan or "nei dan" or "unilateral nostril" or "forced nostril" or "deep breath*" or "martial art*" or taiji or "ai chi" or hatha or asana* or kundalini or bikram or yogi* or ashtanga or "mental heal*" or shavasana or chakra* or mantra* or japa or nirvana or shamatha or tonglen or zen or zazen* or zendo* or bandha* or Dhyana* or dharna or Bhramari or samadhi* or aura or prana* or alaya* or tantra* or "third eye" or vinyasa or lyengar or ashtanga or Bikram or Yin or Bhakti or jnana or tantric or Yama* or niyama* or pranayama* or pratyahara* or "kirtan kriya" or "mind sound resonance technique" or "surya namaskar" or viniyoga or yogafit or "yogasana" or "yog* prana energization technique" or "yog* pranic energization technique" or "wai dan" or waidan or "external elixir" or "internal elixir" or "qi emission" or bhramari or "Nadi shodhana" or pranayama or ujjayi or "kapalabhati" or "kapalbhati" or "anuloma viloma" or "microcosmic orbit" or anusara or "mindfulness-based cognitive therapy" or "cognitive refram*" or "cognitive restructur*" or "intentionally focused aware*")   Results: 871428   1. exp Telemedicine/ or exp Remote Consultation/ or (cyberthera* or telecare or telecollaborat* or teleconsult* or teleconference* or teleeducat* or telediagnos* or telehealth or teleguide* or telediagnos* or telelearn* or telemed* or telementor* or telemonitor* or teleneurol* or teleopth* or telepediatric* or telepresence* or telerehab* or telerobotic* or telescreen* or teletherap* or teletransmi* or mhealth or "m heath" or ehealth* or "e health" or website* or ((cyber or digital or remote* or distance* or tele) adj2 (medicine or care or collaborat* or consult* or conference* or educat* or diagnos* or health or guide* or diagnos* or learn* or med* or mentor* or monitor* or presence* or screen* or therap* or transmi*)) or ((cyber or digital or distance* or tele or remote* or sms or phone* or internet or "web based" or telephone* or texting or "mobile app*" or Instagram or Snapchat or Facetime or GMeet* or hangout* or Skype or Zoom or Web-ex or WebEx or Bluejeans or Facebook or e-mail* or email* or "e chat" or echat or "social media" or "text message*" or "answering machine*" or "voice mail*" or "video conferenc*" or "video link*" or "video chat*") adj3 (consult* or support* or diagnos* or "follow-up*" or "health" or doctor* or "primary care" or clinic or clinics or clinician* or nurs* or psycholog* or therap* or intervention* or delivery))).mp.   Results: 175839   1. fatigue/ or asthenia/ or (fatigu* or physical fatigue or asthenia or cognitive fatigue or mental fatigue or "lack of energy" or tired* or letharg* or malaise or weariness or exhausted or exhaustion or "no energy").mp.   Results: 189729   1. 1 and 2 and 3   Results: 444   1. exp Child/ or "Congenital, Hereditary, and Neonatal Diseases and Abnormalities"/ or exp infant/ or adolescent/ or exp pediatrics/ or child, abandoned/ or exp child, exceptional/ or child, orphaned/ or child, unwanted/ or minors/ or (pediatric* or paediatric* or child* or newborn* or congenital* or infan* or baby or babies or neonat* or pre-term or preterm* or premature birth* or NICU or preschool* or pre-school* or kindergarten* or kindergarden* or elementary school* or nursery school* or (day care* not adult*) or schoolchild* or toddler* or boy or boys or girl* or middle school* or pubescen* or juvenile* or teen* or youth* or high school* or adolesc* or pre-pubesc* or prepubesc*).mp. or (child* or adolesc* or pediat* or paediat*).jn.   Results: 5002496   1. exp adult/ or adult*.mp.   Results: 8559586   1. 4 not (5 not 6)   Results: 408 |
| **PSYCINFO search strategy** |
| 1. mind body therapy/ or exp meditation/ or exp yoga/ or exp relaxation therapy/ or exp mindfulness/ or ("wellness intervention" or "wellness therapy" or "wellness program" or meditat* or mindful* or relaxation or "relaxation therapy" or "breath* exercise*" or "mind body" or "body mind" or "tai chi" or taichi or qigong or breath* or "guided imagery" or "acceptance commitment therapy" or "cognitive behavioural therapy" or "cognitive behavioral therapy" or vitalism or "meditative practice*" or samu or "positive psycholog*" or "intentionally focused awareness" or simonton or visuali?ation or imagery or sophrology or "muscle contract*" or "hold relax" or "alternate nostril" or "abdominal breath*" or nedian or "nei dan" or "unilateral nostril" or "forced nostril" or "deep breath*" or "martial art*" or taiji or "tai ji" or "ai chi" or yoga or hatha or asana* or bikram or yogi* or ashtanga or "mental heal*" or shavasana or chakra* or kundalini or mantra* or japa or nirvana or shamatha or tonglen or zen or zazen* or zendo* or bandha* or Dhyana* or dharna or bhramari or samadhi* or aura* or prana* or alaya* or tantra* or "third eye" or vinyasa or lyengar or Yin or Bhakti or jnana or tantric or Yama* or niyama* or pranayama* or pratyahara* or "kirtan kriya" or "mind sound resonance technique" or "surya namaskar" or viniyoga or yogafit or "yogasana" or "yog* pranic energization technique" or "yog* prana energization technique" or "wai dan" or waidan or "external elixir" or "internal elixir" or "qi emission" or bhramari or "Nadi shodhana" or ujjayi or "kapalabhati" or "kapalbhati" or "anuloma viloma" or "microcosmic orbit" or anusara or "mindful* based cognitive therap*" or "cognitive refram*" or "cognitive restructur*" or "intentional* focused awareness").mp.   Results: 407516   1. exp Digital Interventions/ or electronic health services/ or mobile health/ or exp Telemedicine/ or exp Online Therapy/ or exp Distance Education/ or (((cyberthera* or telecare or telecollaborat* or teleconsult* or teleconference* or teleeducat* or telediagnos* or telehealth or teleguide* or telediagnos* or telelearn* or telemed* or telementor* or telemonitor* or teleneurol* or teleopth* or telepediatric* or telepresence* or telerehab* or telerobotic* or telescreen* or teletherap* or teletransmi* or mhealth or "m heath" or ehealth* or "e health" or (cyber or digital or remote* or distance* or tele)) adj2 (care or collaborat* or consult* or conference* or educat* or diagnos* or health or guide* or diagnos* or learn* or med* or mentor* or monitor* or presence* or screen* or therap* or transmi*)) or ((cyber or digital or distance* or tele or remote* or sms or phone* or internet or "web based" or telephone* or texting or "mobile app*" or Instagram or Snapchat or Facetime or GMeet* or hangout* or Skype or Zoom or Web-ex or WebEx or Bluejeans or Facebook or e-mail* or email* or "e chat" or echat or "social media" or "text message*" or "answering machine*" or "voice mail*" or "video conferenc*" or "video link*" or "video chat*") adj3 (consult* or support* or diagnos* or "follow-up*" or "health" or doctor* or "primary care" or clinic or clinics or clinician* or nurs* or psycholog* or therap* or intervention* or delivery))).mp. [mp=title, abstract, heading word, table of contents, key concepts, original title, tests & measures, mesh word]   Results: 47341   1. fatigue/ or asthenia/ or (fatigu* or physical fatigue or asthenia or cognitive fatigue or mental fatigue or "lack of energy" or tired* or letharg* or malaise or weariness or exhausted or exhaustion or "no energy").mp.   Results: 47128   1. adolescent development/ or childhood development/ or pediatrics/ or exp Congenital Disorders/ or child characteristics/ or child abuse/ or exp child welfare/ or chronically ill children/ or child neglect/ or child psychiatry/ or child psychopathology/ or exp child care/ or (pediatric* or paediatric* or child* or newborn* or congenital* or infan* or baby or babies or neonat* or pre term or preterm* or premature birth or NICU or preschool* or pre school* or kindergarten* or elementary school* or nursery school* or schoolchild* or toddler* or boy or boys or girl* or middle school* or pubescen* or juvenile* or teen* or youth* or high school* or adolesc* or prepubesc* or pre pubesc*).mp. or (child* or adolesc* or pediat* or paediat*).jn.   Results: 1413468   1. exp adult attitudes/ or exp adult development/ or exp adult education/ or exp adult learning/ or adult*.mp.   Results: 1042230   1. 1 and 2 and 3   Results: 159   1. 6 not (4 not 5)   Results: 140 |
| **CINAHL search strategy** |
| 1. (MH "Yoga+") or (MH "Tai Chi") or (MH "Meditation") or (MH "Breathing Exercises+") or (MH "Qigong") or (MH "Mindfulness+") or ("wellness intervention" or "wellness therapy" or "wellness program" or meditat* or mindful* or relaxation or "relaxation therapy" or "breath* exercise*" or "mind body" or "body mind" or "tai chi" or taichi or qigong or breath* or "guided imagery" or "acceptance commitment therapy" or "cognitive behavioural therapy" or "cognitive behavioral therapy" or vitalism or "meditative practice*" or samu or "positive psycholog*" or "intentionally focused awareness" or simonton or visuali?ation or imagery or sophrology or "muscle contract*" or "hold relax" or "alternate nostril" or "abdominal breath*" or nedian or "nei dan" or "unilateral nostril" or "forced nostril" or "deep breath*" or "martial art*" or taiji or "tai ji" or "ai chi" or yoga or hatha or asana* or bikram or yogi* or ashtanga or "mental heal*" or shavasana or chakra* or kundalini or mantra* or japa or nirvana or shamatha or tonglen or zen or zazen* or zendo* or bandha* or Dhyana* or dharna or bhramari or samadhi* or aura* or prana* or alaya* or tantra* or "third eye" or vinyasa or lyengar or Yin or Bhakti or jnana or tantric or Yama* or niyama* or pranayama* or pratyahara* or "kirtan kriya" or "mind sound resonance technique" or "surya namaskar" or viniyoga or yogafit or "yogasana" or "yog* pranic energization technique" or "yog* prana energization technique" or "wai dan" or waidan or "external elixir" or "internal elixir" or "qi emission" or bhramari or "Nadi shodhana" or ujjayi or "kapalabhati" or "kapalbhati" or "anuloma viloma" or "microcosmic orbit" or anusara or "mindful* based cognitive therap*" or "cognitive refram*" or "cognitive restructur*" or "intentional* focused awareness")   Results: 379,053   1. (MH "Telehealth+") OR (MH "Telemedicine+") OR (MH "Remote Consultation") OR (MH "Telerehabilitation") OR (MH "Telenursing") OR (MH "Telenutrition") OR (MH "Telepsychiatry") or (((cyberthera* or telecare or telecollaborat* or teleconsult* or teleconference* or teleeducat* or telediagnos* or telehealth or teleguide* or telediagnos* or telelearn* or telemed* or telementor* or telemonitor* or teleneurol* or teleopth* or telepediatric* or telepresence* or telerehab* or telerobotic* or telescreen* or teletherap* or teletransmi* or mhealth or "m heath" or ehealth* or "e health" or (cyber or digital or remote* or distance* or tele)) N2 (care or collaborat* or consult* or conference* or educat* or diagnos* or health or guide* or diagnos* or learn* or med* or mentor* or monitor* or presence* or screen* or therap* or transmi*)) or ((cyber or digital or distance* or tele or remote* or sms or phone* or internet or "web based" or telephone* or texting or "mobile app*" or Instagram or Snapchat or Facetime or GMeet* or hangout* or Skype or Zoom or Web-ex or WebEx or Bluejeans or Facebook or e-mail* or email* or "e chat" or echat or "social media" or "text message*" or "answering machine*" or "voice mail*" or "video conferenc*" or "video link*" or "video chat*") N3 (consult* or support* or diagnos* or "follow-up*" or "health" or doctor* or "primary care" or clinic or clinics or clinician* or nurs* or psycholog* or therap* or intervention* or delivery)))   Results: 78,946   1. (MH "fatigue+") or (MH "asthenia") or (fatigu* or physical fatigue or asthenia or cognitive fatigue or mental fatigue or "lack of energy" or tired* or letharg* or malaise or weariness or exhausted or exhaustion or "no energy")   Results : 68,476   1. S1 AND S2 AND S3   Results: 177   1. (pediatric* or paediatric* or child* or newborn* or congenital* or infan* or baby or babies or neonat* or “pre-term” or preterm or “premature birth*” or NICU or preschool* or “pre-school*” or kindergarten* or “elementary school*” or “nursery school*” or schoolchild* or toddler* or boy or boys or girl* or “middle school*” or pubescen* or juvenile* or teen* or youth* or “high school*” or adolesc*or prepubesc* or “pre-pubesc*” or (MH "Child+") OR (MH "Adolescence+") OR (MH "Minors (Legal)") or "(MH "Child Abuse, Sexual") OR (MH "Child Behavior Disorders+") OR (MH "Child, Medically Fragile") OR (MH "Child Day Care") OR (MH "Child Behavior+") OR (MH "Child Mortality") OR (MH "Child Passenger Safety") OR (MH "Child Development Disorders, Pervasive+") OR (MH "Child Custody") OR (MH "Child Abuse+") OR (MH "Child Nutritional Physiology+") OR (MH "Child Behavior Checklist") ) OR SO ( child* or pediatric* or paediatric* or adolescent* )   Results: 1,532,669   1. (MH "Adult+") OR "adult*"   Results: 2,185,777   1. S4 NOT (S5 NOT S6)   Results: 165 |
| **EMBASE search strategy** |
| 1. exp mindfulness/ or exp meditation/ or exp breathing exercise/ or exp yoga/ or exp tai chi/ or exp relaxation training/ or ("meditat*" or "wellness intervention" or "wellness therapy" or "wellness program" or mindful* or relaxation or "relaxation therapy" or yoga or "tai ji" or qigong or "breath* exercises" or mind-body or body-mind or "tai chi" or taichi or qigong or breath* or "guided imagery" or "acceptance commitment therapy" or "cognitive behavioural therapy" or "cognitive behavioral therapy" or vitalism or "meditative practice" or samu or "positive psycholog*" or "intentionally focused awareness" or simonton or visuali?ation or imagery or sophrology or "muscle contract*" or "hold relax" or "alternate nostril" or "abdominal breath" or neidan or "nei dan" or "unilateral nostril" or "forced nostril" or "deep breath*" or "martial art*" or taiji or "ai chi" or hatha or asana* or kundalini or bikram or yogi* or ashtanga or "mental heal*" or shavasana or chakra* or mantra* or japa or nirvana or shamatha or tonglen or zen or zazen* or zendo* or bandha* or Dhyana* or dharna or Bhramari or samadhi* or aura or prana* or alaya* or tantra* or "third eye" or vinyasa or lyengar or ashtanga or Bikram or Yin or Bhakti or jnana or tantric or Yama* or niyama* or pranayama* or pratyahara* or "kirtan kriya" or "mind sound resonance technique" or "surya namaskar" or viniyoga or yogafit or "yogasana" or "yogic prana energization technique" or "yog* pranic energization technique or wai dan" or waidan or "external elixir" or "internal elixir" or "qi emission" or bhramari or "Nadi shodhana" or pranayama or ujjayi or "kapalabhati" or "kapalbhati" or "anuloma viloma" or "microcosmic orbit" or anusara or "mindfulness-based cognitive therapy" or "cognitive refram*" or "cognitive restructur*" or "intentionally focused aware*").mp. [mp=title, abstract, heading word, drug trade name, original title, device manufacturer, drug manufacturer, device trade name, keyword heading word, floating subheading word, candidate term word]   Results: 1304516   1. exp telemedicine/ or exp teleconsultation/ or (((cyberthera* or telecare or telecollaborat* or teleconsult* or teleconference* or teleeducat* or telediagnos* or telehealth or teleguide* or telediagnos* or telelearn* or telemed* or telementor* or telemonitor* or teleneurol* or teleopth* or telepediatric* or telepresence* or telerehab* or telerobotic* or telescreen* or teletherap* or teletransmi* or mhealth or "m heath" or ehealth* or "e health" or (cyber or digital or remote* or distance* or tele)) adj2 (care or collaborat* or consult* or conference* or educat* or diagnos* or health or guide* or diagnos* or learn* or med* or mentor* or monitor* or presence* or screen* or therap* or transmi*)) or ((cyber or digital or distance* or tele or remote* or sms or phone* or internet or "web based" or telephone* or texting or "mobile app*" or Instagram or Snapchat or Facetime or GMeet* or hangout* or Skype or Zoom or Web-ex or WebEx or Bluejeans or Facebook or e-mail* or email* or "e chat" or echat or "social media" or "text message*" or "answering machine*" or "voice mail*" or "video conferenc*" or "video link*" or "video chat*") adj3 (consult* or support* or diagnos* or follow-up* or health or doctor* or "primary care" or clinic or clinics or clinician* or nurs* or psycholog* or therap* or intervention* or delivery))).mp. [mp=title, abstract, heading word, drug trade name, original title, device manufacturer, drug manufacturer, device trade name, keyword heading word, floating subheading word, candidate term word]   Results: 163137   1. fatigue/ or asthenia/ or (fatigu* or physical fatigue or asthenia or cognitive fatigue or mental fatigue or "lack of energy" or tired* or letharg* or malaise or weariness or exhausted or exhaustion or "no energy").mp.   Results: 448459   1. juvenile/ or exp adolescent/ or exp child/ or exp postnatal development/ or (pediatric* or paediatric* or child* or newborn* or congenital* or infan* or baby or babies or neonat* or pre term or preterm* or premature birth or NICU or preschool* or pre school* or kindergarten* or elementary school* or nursery school* or schoolchild* or toddler* or boy or boys or girl* or middle school* or pubescen* or juvenile* or teen* or youth* or high school* or adolesc* or prepubesc* or pre pubesc*).mp. or (child* or adolesc* or pediat* or paediat*).jn.   Results: 5267434   1. exp adult/ or adult*.mp. [mp=title, abstract, heading word, drug trade name, original title, device manufacturer, drug manufacturer, device trade name, keyword heading word, floating subheading word, candidate term word]   Results: 10745933   1. 1 and 2 and 3   Results: 772   1. 6 not (4 not 5)   Results: 715 |
| **SCOPUS search strategy** |
| ( TITLE-ABS-KEY ( "mind-body therapies" OR "breathing exercises" OR meditation OR meditat* OR "wellness intervention" OR "wellness therapy" OR "wellness program" OR mindful* OR relaxation OR "relaxation therapy" OR yoga OR "tai ji" OR qigong OR "breath* exercises" OR "mind-body" OR "body-mind" OR "tai chi" OR taichi OR qigong OR breath* OR "guided imagery" OR "acceptance commitment therapy" OR "cognitive behavioural therapy" OR "cognitive behavioral therapy" OR vitalism OR "meditative practice" OR samu OR "positive psycholog*" OR "intentionally focused awareness" OR simonton OR visuali?ation OR imagery OR sophrology OR "muscle contract*" OR "hold relax" OR "alternate nostril" OR "abdominal breath" OR neidan OR "nei dan" OR "unilateral nostril" OR "forced nostril" OR "deep breath*" OR "martial art*" OR taiji OR "ai chi" OR hatha OR asana* OR kundalini OR bikram OR yogi* OR ashtanga OR "mental heal*" OR shavasana OR chakra* OR mantra* OR japa OR nirvana OR shamatha OR tonglen OR zen OR zazen* OR zendo* OR bandha* OR dhyana* OR dharna OR bhramari OR samadhi* OR aura OR prana* OR alaya* OR tantra* OR "third eye" OR vinyasa OR lyengar OR ashtanga OR bikram OR yin OR bhakti OR jnana OR tantric OR yama* OR niyama* OR pranayama* OR pratyahara* OR "kirtan kriya" OR "mind sound resonance technique" OR "surya namaskar" OR viniyoga OR yogafit OR "yogasana" OR "yog* prana energization technique" OR "yog* pranic energization technique" OR "wai dan" OR waidan OR "external elixir" OR "internal elixir" OR "qi emission" OR bhramari OR "Nadi shodhana" OR pranayama OR ujjayi OR "kapalabhati" OR "kapalbhati" OR "anuloma viloma" OR "microcosmic orbit" OR anusara OR "mindfulness-based cognitive therapy" OR "cognitive refram*" OR "cognitive restructur*" OR "intentionally focused aware*" ) AND TITLE-ABS-KEY ( telemedicine OR "remote consultation" OR cyberthera* OR telecare OR telecollaborat* OR teleconsult* OR teleconference* OR teleeducat* OR telediagnos* OR telehealth OR teleguide* OR telediagnos* OR telelearn* OR telemed* OR telementor* OR telemonitor* OR teleneurol* OR teleopth* OR telepediatric* OR telepresence* OR telerehab* OR telerobotic* OR telescreen* OR teletherap* OR teletransmi* OR mhealth OR "m heath" OR ehealth* OR "e health" OR website* OR ( ( cyber OR digital OR remote* OR distance* OR tele ) W/2 ( medicine OR care OR collaborat* OR consult* OR conference* OR educat* OR diagnos* OR health OR guide* OR diagnos* OR learn* OR med* OR mentor* OR monitor* OR presence* OR screen* OR therap* OR transmi* ) ) OR ( ( cyber OR digital OR distance* OR tele OR remote* OR sms OR phone* OR internet OR "web based" OR telephone* OR texting OR "mobile app*" OR instagram OR snapchat OR facetime OR gmeet* OR hangout* OR skype OR zoom OR web-ex OR webex OR bluejeans OR facebook OR e-mail* OR email* OR "e chat" OR echat OR "social media" OR "text message*" OR "answering machine*" OR "voice mail*" OR "video conferenc*" OR "video link*" OR "video chat*" ) W/3 ( consult* OR support* OR diagnos* OR "follow-up*" OR "health" OR doctor* OR "primary care" OR clinic OR clinics OR clinician* OR nurs* OR psycholog* OR therap* OR intervention* OR delivery ) ) ) AND TITLE-ABS-KEY ( fatigue OR asthenia OR fatigu* OR "physical fatigue" OR "cognitive fatigue" OR "mental fatigue" OR "lack of energy" OR tired* OR letharg* OR malaise OR weariness OR exhausted OR exhaustion OR "no energy" ) ) AND NOT ( ( TITLE-ABS-KEY ( pediatric* OR paediatric* OR child OR children OR newborn* OR congenital* OR infan* OR baby OR babies OR neonat* OR "pre-term" OR preterm OR "premature birth*" OR nicu OR preschool* OR "pre-school*" OR kindergarten* OR "elementary school*" OR "nursery school*" OR schoolchild* OR toddler* OR boy OR boys OR girl* OR "middle school*" OR pubescen* OR juvenile* OR teen* OR youth* OR "high school*" OR adolesc* OR prepubesc* OR "pre-pubesc*" ) OR SRCTITLE ( child OR children OR pediatric* OR paediatric* OR adolescent ) ) AND NOT TITLE-ABS-KEY ( adult* ) ) |
| **COCHRANE search strategy** |
| 1. [mh "mind body therapies"] or [mh "breathing exercises"] or [mh "meditation"] or ("wellness intervention" or "wellness therapy" or "wellness program" or meditat* or mindful* or relaxation or "relaxation therapy" or "breath* exercise*" or "mind body" or "body mind" or "tai chi" or taichi or qigong or breath* or "guided imagery" or "acceptance commitment therapy" or "cognitive behavioural therapy" or "cognitive behavioral therapy" or vitalism or "meditative practice*" or samu or "positive psycholog*" or "intentionally focused awareness" or simonton or visuali?ation or imagery or sophrology or "muscle contract*" or "hold relax" or "alternate nostril" or "abdominal breath*" or nedian or "nei dan" or "unilateral nostril" or "forced nostril" or "deep breath*" or "martial art*" or taiji or "tai ji" or "ai chi" or yoga or hatha or asana* or bikram or yogi* or ashtanga or "mental heal*" or shavasana or chakra* or kundalini or mantra* or japa or nirvana or shamatha or tonglen or zen or zazen* or zendo* or bandha* or Dhyana* or dharna or bhramari or samadhi* or aura* or prana* or alaya* or tantra* or "third eye" or vinyasa or lyengar or Yin or Bhakti or jnana or tantric or Yama* or niyama* or pranayama* or pratyahara* or "kirtan kriya" or "mind sound resonance technique" or "surya namaskar" or viniyoga or yogafit or "yogasana" or "yog* pranic energization technique" or "yog* prana energization technique" or "wai dan" or waidan or "external elixir" or "internal elixir" or "qi emission" or bhramari or "Nadi shodhana" or ujjayi or "kapalabhati" or "kapalbhati" or "anuloma viloma" or "microcosmic orbit" or anusara or "mindful* based cognitive therap*" or "cognitive refram*" or "cognitive restructur*" or "intentional* focused awareness"):ti,ab,kw 2. [mh "Telemedicine"] or [mh "Remote Consultation"] or (cyberthera* or telecare or telecollaborat* or teleconsult* or teleconference* or teleeducat* or telediagnos* or telehealth or teleguide* or telediagnos* or telelearn* or telemed* or telementor* or telemonitor* or teleneurol* or teleopth* or telepediatric* or telepresence* or telerehab* or telerobotic* or telescreen* or teletherap* or teletransmi* or mhealth or "m heath" or ehealth* or "e health" or website* or ((cyber or digital or remote* or distance* or tele) NEAR/2 (medicine or care or collaborat* or consult* or conference* or educat* or diagnos* or health or guide* or diagnos* or learn* or med* or mentor* or monitor* or presence* or screen* or therap* or transmi*)) or ((cyber or digital or distance* or tele or remote* or sms or phone* or internet or "web based" or telephone* or texting or "mobile app*" or Instagram or Snapchat or Facetime or GMeet* or hangout* or Skype or Zoom or Web-ex or WebEx or Bluejeans or Facebook or e-mail* or email* or "e chat" or echat or "social media" or "text message*" or "answering machine*" or "voice mail*" or "video conferenc*" or "video link*" or "video chat*") NEAR/3 (consult* or support* or diagnos* or "follow-up*" or "health" or doctor* or "primary care" or clinic or clinics or clinician* or nurs* or psycholog* or therap* or intervention* or delivery))):ti,ab,kw 3. [mh "fatigue"] or [mh "asthenia"] or (fatigu* or "physical fatigue" or asthenia or "cognitive fatigue" or "mental fatigue" or "lack of energy" or tired* or letharg* or malaise or weariness or exhausted or exhaustion or "no energy"):ti,ab,kw 4. #1 AND #2 AND #3 5. [mh "Child"] or ([mh "Congenital, Hereditary and Neonatal Diseases and Abnormalities"]) or [mh "infant"] or [mh ^"adolescent"] or [mh "pediatrics"] or [mh ^"child, abandoned"] or [mh "child, exceptional"] or [mh ^"child, orphaned"] or [mh ^"child, unwanted"] or [mh ^"minor"] or (pediatric* or paediatric* or child* or newborn* or congenital* or infan* or baby or babies or neonat* or pre-term or preterm* or (premature NEXT birth) or NICU or preschool* or (pre NEXT school*) or kindergarten* or kindergarden* or (elementary NEXT school*) or (nursery NEXT school*) or ((day NEXT care*) not adult*) or schoolchild* or toddler* or boy or boys or girl* or (middle NEXT school*) or pubescen* or juvenile* or teen* or youth* or (high NEXT school*) or adolesc* or (pre NEXT pubesc*) or prepubesc*):ti,ab,kw or (child* or adolesc* or pediat* or paediat*):so 6. [mh adult] or adult*:ti,ab,kw 7. #4 NOT (#5 NOT #6) |
