# Supplementary material for: The impact of mind-body internet and mobile-based interventions on fatigue in adults living with chronic physical conditions: A systematic review and meta-analysis of randomized controlled trials
Source: PLOS Digit Health. 2025 Jun 11;4(6):e0000878. doi: 10.1371/journal.pdig.0000878 (PMC12157242; doi:10.1371/journal.pdig.0000878)
Supplement: S6 Appendix — Adherence data. (DOCX) [file pdig.0000878.s006.docx]

| Author (Year) | Adherence data collected (Y/N) | Method of collecting adherence Data | Adherence data |
| --- | --- | --- | --- |
| Boele et al. (2018) [53] | Y | Completion rates for the introduction and module(s) 1-5 | Introduction = 85%  Module 1 = 77%  Module 2 = 52%  Module 3 = 40%  Module 4 = 37%  Module 5 = 35% |
| Ferwerda et al. (2017) [66] | N | NI | NI |
| Fischer et al. (2015) [58] |  | Retrospective participant self-appraisal of using the intervention program ‘regularly preforming exercises over the past weeks’ | 77.1% |
| Friesen et al. (2017) [62] | Y | (1) The average amount of times participants accessed the program;(2) average minutes participants spent on the phone with personnel (adherence to calls) | (1) 23.43 times (SD=13.85);  (2)18.5 minutes (SD=13.21) |
| Huberty et al. (2019) [54] | Y | Average time (minutes) participants accessed the program per week recorded by the (1) program and (2) self-reported by participants | (1) 40.8 minutes/week; (2) 56.1 minutes/week |
| İşcan Ayyildiz et al. (2024)[65] | Y | The “length of stay” participants had on the web platform | NI |
| Kubo et al. (2019) [55] | Y | Assessed the number and percent of participants that used the program (1) at least 50%, and (2) 70% of the 8-week study period, (3) continued program use after 8-week study period | (1) 50%  (2) 32.5%  (3) 70% |
| Menting et al. (2017) [68] |  | (1) 11-point Likert scale (0 – not adherent at all, 10- fully adherent) completed by the therapist at the end of study and, (2) 5-point Likert scale of 1 (not adherent at all) to 5 (fully adherent), filled in by the patient for each module, (3) mean time spent on the website | (1) 7.4 (SD=1.4)  (2) 3.4 (SD=0.8)  (3) 5 hours and 33 minutes |
| Moss-Morris et al. (2012) [59] | Y | (1) Mean number of completed sessions assessed at week 10, (2) number and percent of participants that completed more than half the sessions, (3) participants that completed each telephone session 1, 2 and 3 | (1) 4.91 (SD = 2.10)  (2) 60.8%;  (3) 91%,82%, 65% |
| Neubert et al. (2023) [56] | Y | Percent of participants that (1) watched video sequences, (2) watched videos multiple times (less than or equal to 2 times, 3-5 times and more than 5 times), (3) exercised during the intervention (no, partly, 1-2 times a week, 3-5 times a week and daily) | (1) 83%  (2) 51%,15%,2%  (3)8%,42%, 28%, 19%,3% |
| Pöttgen et al. (2018) [60] | Y | Average amount of times participants accessed the program | 14.5 times (SD=13) |
| Schröder et al. (2014) [64] | N | NI | NI |
| Titcomb et al. (2023) [61] | Y | (1) Number of participants who completed the program orientation, (2) Percent of participants who completed modules 1-5 (numeric values were only provided for modules 1 and 5 for the total group – intervention and WLC, graphical data shows completion rate for all modules according to each arm and the combined arms). | (1) n=96  (2) Intervention group. Module 1: ~91%, Module 2: ~80%, Module 3: ~65%, Module 4: ~60%, Module 5: 49% |
| Urech et al. (2018) [57] | Y | Percent of participants that accessed at least 6 of 8 modules | 80% |
| van Beugen et al. (2016) [67] | Y | Assessed by (1) patient self-report of adherence, (2) therapist rating of adherence and (3) intervention website use | NI |
| Watt et al. (2023)[69] | Y | Number of times in week participants completed the video routine (the research group recommended | <1 time/week: n=3  1 time/week: n=7  2 times/week: n=6  3 times/week: n=10  4 times/week: n=3  5 times/week: n=3  6 times/week: n=3  7 times/week: n=2 |
| Williams et al. (2010) [63] |  | Assessed on a monthly basis and at follow-up, adherence was recorded through the average number of skills participants used from the program each month | 1 module was being used each month by 89-94% of the sample.  Month 1: 3.7 skills used  Month 2: 4.4 skills used  Month 3: 4.2 skills used  Month 4: 4.2 skills used  Month 5: 4.2 skills used  Month 6: 4.4 skills used |

Y = Yes; N = No, NI = No Information
