# Supplementary material for: The impact of mind-body internet and mobile-based interventions on fatigue in adults living with chronic physical conditions: A systematic review and meta-analysis of randomized controlled trials
Source: PLOS Digit Health. 2025 Jun 11;4(6):e0000878. doi: 10.1371/journal.pdig.0000878 (PMC12157242; doi:10.1371/journal.pdig.0000878)
Supplement: S7 Appendix — Demographic data. (DOCX) [file pdig.0000878.s007.docx]

| **Author (Year)** | **Education** | **Income** | **Employment** | **Ethnicity** | **Sex/Gender Reports** | **Marital Status** | **Technology**  **Proficiency** |
| --- | --- | --- | --- | --- | --- | --- | --- |
| Boele et al. (2018) [53] | Low =8 (8.8%) Middle = 36 (46.3%) High = 38 (46.3%) | NI | NI | NI | Female: 45 (54.9%)  Male: 37 (45.1%) | NI | NI |
| Ferwerda et al. (2017) [66] | Primary= 2 (2%) Secondary=97 (73%) Tertiary= 34 (26%) | NI | NI | NI | Female: 85 (64%)  Male: 48 (36%) | NI | User friendliness', data retrieved for 33 participants, mean score=7.09 (range= 1-10) (SD=1.89) |
| Fischer et al. (2015) [58] | Education in years 9yrs - 13 (14.4%) 10yrs - 35 (38.9%) 13yrs - 42 (46.7%) | NI | NI | NI | Female: 70 (77.8%)  Male: 20 (22.2%) | NI | NI |
| Friesen et al. (2017) [62] | Less than high school -2 (3%) High school diploma 13 (22%) College certificate or diploma 24 (40%) Some University 9 (15%) University Degree 12 (20%) | NI | Employed full-time or part time 16 (26%) Unemployed or retired 9 (15%) On short-term or long-term disability 27 (45%) Homemaker 6 (10%) Student 2 (3%) | White/Caucasian 57 (95%) Spanish/Hispanic/Latino 1 (2%) Mixed Ethnicity 2 (3%) | Female: 57 (95%)  Male: 3 (5%) | Marital status Single, Never Married ­­­­10 (17%) Married 37 (62%) Living with Partner 5 (8%) Divorced 5 (8%) Widowed 3 (5%) | End-of-Study Qualitative reports about the content and user-friendliness of the course. |
| Huberty et al. (2019) [54] | < High School 0 (0%) High school diploma 2 (4.2%) Some college 3 (6.3%) Associates/2-year degree 3 (6.3%) Bachelors degree 18 (37.5%)  Graduate school or above 22 (45.8%) | NI | NI | Caucasian 45 (93.8%) Other 3 (6.3%) | Female: 45 (93.8%%)  Male: 3 (6.3%) | Single 2 (4.2%) Partnered/in a relationship 3 (6.3%) Married 41 (85.4%) Separated 41 (85.4%) Divorced | NI |
| İșcan Ayyildiz et al. (2024) [65] | Literate: 2 (3.3%), Primary School: 15 (25%), Highschool: 33 (55%), Graduate and post-graduate: 10 (16.6%) | Income less than expenses: 48 (80%), Income equal to expenses: 12 (20%) | Housewife: 23 (38.3%), Officer: 6 (10%), Employee: 5 (8.3%), Student: 13 (21.7%), Retired: 9 (15%), Unemployed: 4 (6.7%) | NI | Female: 30 (50%)  Male: 30 (50%) | Married: 36 (60%), Single: 34 (40%) | NI |
| Kubo et al. (2019) [55] | Some college or less 37 (38%) College graduate 33 (34%) Postgraduate degree 26 (27%) | Less than $75 000 - 32 (33%) $75 000 to $99 999 -12 (12.4%) $100 000 to $149 999 - 26 (27%) $150 000 or more - 17 (18%) | NI | White 63 (65%) African American 6 (6%) Asian 7 (7%) Other 17 (18%) Unknown/not reported 4 (4%) | Female: 66 (68%)  Male:31 (32%) | Married 61 (63%) Living as married/domestic partner 3 (3%) Widowed 4 (4%) Separated/divorced 15 (15%) Never married 10 (10%) | NI |
| Menting et al. (2017) [68] | Low 18 (15%) Medium 61 (51%)  High 41 (24%) | NI | NI | NI | Female: 74 (62%)  Male: 46 (38%) | NI | NI |
| Moss-Morris et al. (2012) [59] | NI | NI | Working less or off sick 3 (7.5%) Unemployed 11 (28%) | NI | Female: 32 (80%)  Male: 8 (205) | Single 8 (20%) Living with partner 22 (55%) Divorced/widowed 8 (20%) Missing data 2 (5%) | End-of-study qualitative interviews. (n=15) |
| Neubert et al. (2023) [56] | NI | NI | NI | NI | Female: 108 (69%)  Male: 49 (31%) | Physical, cognitive and emotional aspects | NI |
| Pöttgen et al. (2018) [60] | Education in years  Mean: 14.76 (SD=2.16) | NI | Full-time/part-time employment 135 (49%) Housemaker/student/other 24 (9%) Unemployed/unable to work/retired 116 (42%) | NI | Female: 222 (81%)  Male: 53 (19%) | Married/living with partner 196 (71%) Single/divorced/widowed 79 (29%) | NI |
| Schröder et al. (2014) [64] | Education Completed Highschool 37 (47%) Did not complete Highschool 41 (53%) | NI | NI | NI | Female: 59 (76%)  Male: 19 (24%) | NI | NI; 1 participant was lost due to technical issues with the program |
| Titcomb et al. (2023) [61] | NI | NI | NI | White: 40 (81.6%), Black: 2 (6.1%), Asian: 2 (4.1%), Middle East or North African: 1 (2.0%), American Indian or Alaskan Native: 1 (2.0%), Latin or Hispanic: 0 (0%), Unknown or not reported: 2 (4.1%) | Female: 94 (94%)  Male: 6 (6%) | NI | NI |
| Urech et al. (2018) [57] | Compulsory school 2 (1.6%) Apprenticeship 32 (24.8%) College 44 (34.1%) University 48 (37.2%)  Other 3 (2.3%) | n=107 in euro <1,200 : 2 (1.9%) 1,200-2,500 : 7 (6.5%) 2,500-4,900 : 35 (32.7%) 4,900-8,100 : 36 (33.6%) 8,100-12,200 : 19 (17.8%) >12,200 : 8 (7.5%) | NI | NI | Female: 109 (85%)  Male: 20 (15%) | Married 81 (62.8%) Married, separated 2 (1.6%) Single 26 (20.2%) Divorced 16 (12.4%) Widowed 4 (3.1%) | NI |
| van Beugen et al. (2016) [67] | Primary 1 (1.5%) Secondary 44 (67.7%) Tertiary 20 (30.8%) | NI | NI | NI | Female: 64 (48%)  Male: 67 (52%) | Married/living together 46 (70.8%) | NI |
| Watt et al. (2023) [69] | No post secondary degree certificate, or diploma: 9 (20.9%), Trade certificate or diploma from a vocational school of apprenticeship training: 1 (2.3%), Non-university certificate or diploma from a vocational school of apprenticeship training: : 7(16.3%), University certificate below bachelor's level: 2 (4.7%), Bachelor's degree: 9 (20.9%), University degree or certificate above bachelor's degree: 13 (30.2%), Unknown: 2 (4.7%) | NI | Employed: 40 (46.0%), Unemployed: 34 (39.1%), Prefer not to answer: 13 (14.9%) | NI | Female: 85 (98%)  Male: 2 (2%) | Married: 55 (63.2%), Living common-law: 7 (8.0%), Divorced/separated: 14 (16.1%), Widowed: 4 (4.6%). Single/never married: 5 (5.7%), Prefer not to answer: 2 (2.3%) | NI |
| Williams et al. (2010) [63] | Post graduate degree 14 (12%)  College graduate 35 (30%)  Some college 47 (40%)  High school or less 22 (18%) | NI | NI | White 115 (97%) Other 3 (3%) | Female: 112 (95%)  Male: 6 (5%) | Single 13 (11%)  Married 93 (79%)  Separated, widowed, divorced 12 (10%) | NI |

Y = Yes; N = No, NI = No Information
