# Supplementary material for: The impact of mind-body internet and mobile-based interventions on fatigue in adults living with chronic physical conditions: A systematic review and meta-analysis of randomized controlled trials
Source: PLOS Digit Health. 2025 Jun 11;4(6):e0000878. doi: 10.1371/journal.pdig.0000878 (PMC12157242; doi:10.1371/journal.pdig.0000878)
Supplement: Supplementary file 5 [file pdig.0000878.s002.docx]

Study or Subgroup	Experimental Mean	Experimental SD	Experimental Total	Control Mean	Control SD	Control Total	Weight	Std. Mean Difference IV, Random, 95% CI
**4.1.1 CIS**								
Ferwerda 2017	−3.85	11.47502941	46	−2.36	10.40024519	59	33.6%	−0.14 [−0.52, 0.25]
Menting 2017	−19.4	10.04639239	60	−5.6	9.02662728	60	33.3%	−1.44 [−1.84, −1.03]
van Beugen 2016	−6.93	11.67224057	44	−0.73	9.79229799	49	33.1%	−0.57 [−0.99, −0.16]
**Subtotal (95% CI)**			**150**			**168**	**100.0%**	**−0.71 [−1.47, 0.04]**
Heterogeneity: $Tau^2 = 0.41$; $Chi^2 = 21.39$, df = 2 (P < 0.0001); $I^2 = 91\%$								
Test for overall effect: Z = 1.85 (P = 0.06)								
**4.1.2 MFIS**								
Moss-Morris 2012	−4.17	3.78035713	21	0.19	3.89	16	32.5%	−1.11 [−1.82, −0.41]
Titcomb 2023	−5.1	2.27651049	46	1.3	2.51573051	49	33.4%	−2.64 [−3.20, −2.09]
Watt 2023	−2.7	16.20833119	43	0.8	15.91508718	44	34.0%	−0.22 [−0.64, 0.21]
**Subtotal (95% CI)**			**110**			**109**	**100.0%**	**−1.32 [−2.85, 0.21]**
Heterogeneity: $Tau^2 = 1.74$; $Chi^2 = 46.44$, df = 2 (P < 0.00001); $I^2 = 96\%$								
Test for overall effect: Z = 1.69 (P = 0.09)								
**4.1.3 FSS**								
İşcan Ayyildiz 2024	−1.42	0.7332803	30	0.08	0.65023073	30	49.4%	−2.14 [−2.78, −1.49]
Titcomb 2023	−0.68	0.21517435	46	0.04	0.24062419	49	50.6%	−3.12 [−3.73, −2.52]
**Subtotal (95% CI)**			**76**			**79**	**100.0%**	**−2.64 [−3.60, −1.67]**
Heterogeneity: $Tau^2 = 0.39$; $Chi^2 = 4.80$, df = 1 (P = 0.03); $I^2 = 79\%$								
Test for overall effect: Z = 5.34 (P < 0.00001)								
**4.1.4 Chalder**								
Moss-Morris 2012	−9	5.98862255	20	−1.96	4.61740187	16	41.7%	−1.27 [−2.00, −0.54]
Pöttgen 2018	−2.74	6.7	139	0	6.7	136	58.3%	−0.41 [−0.65, −0.17]
**Subtotal (95% CI)**			**159**			**152**	**100.0%**	**−0.77 [−1.60, 0.06]**
Heterogeneity: $Tau^2 = 0.29$; $Chi^2 = 4.86$, df = 1 (P = 0.03); $I^2 = 79\%$								
Test for overall effect: Z = 1.81 (P = 0.07)								
**4.1.5 FMSC**								
Fischer 2015	−4.03	14.94901669	35	−0.97	15.65690902	36	20.7%	−0.20 [−0.66, 0.27]
Pöttgen 2018	−3.74	9.8	139	0	9.8	136	79.3%	−0.38 [−0.62, −0.14]
**Subtotal (95% CI)**			**174**			**172**	**100.0%**	**−0.34 [−0.56, −0.13]**
Heterogeneity: $Tau^2 = 0.00$; $Chi^2 = 0.47$, df = 1 (P = 0.49); $I^2 = 0\%$								
Test for overall effect: Z = 3.16 (P = 0.002)								
**4.1.6 HAQUAMS**								
Fischer 2015	−0.19	1.03014562	35	0.01	1.0705606	36	26.5%	−0.19 [−0.65, 0.28]
Pöttgen 2018	−0.39	0.8	139	0	0.8	136	73.5%	−0.49 [−0.73, −0.25]
**Subtotal (95% CI)**			**174**			**172**	**100.0%**	**−0.41 [−0.66, −0.15]**
Heterogeneity: $Tau^2 = 0.01$; $Chi^2 = 1.24$, df = 1 (P = 0.27); $I^2 = 19\%$								
Test for overall effect: Z = 3.09 (P = 0.002)								

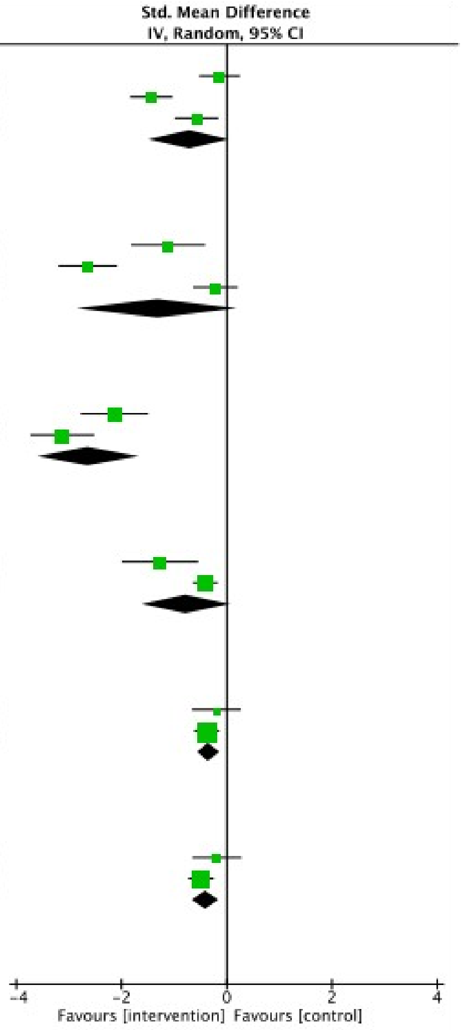

Test for subgroup differences: $Chi^2 = 22.94$, df = 5 (P = 0.0003), $I^2 = 78.2\%$
