# Supplementary material for: The impact of mind-body internet and mobile-based interventions on fatigue in adults living with chronic physical conditions: A systematic review and meta-analysis of randomized controlled trials
Source: PLOS Digit Health. 2025 Jun 11;4(6):e0000878. doi: 10.1371/journal.pdig.0000878 (PMC12157242; doi:10.1371/journal.pdig.0000878)

Study or Subgroup	Experimental Mean	SD	Total	Control Mean	SD	Total	Weight	Std. Mean Difference IV, Random, 95% CI
**2.12.1 Cancer**								
Boele 2018	−6.69	24.12550518	19	−0.42	17.44505661	33	5.7%	−0.31 [−0.87, 0.26]
Huberty 2019	−0.5	2.2	27	−0.6	2.3	21	5.6%	0.04 [−0.53, 0.61]
Kubo 2019	−0.5	2.45153013	40	0	2.2	32	5.9%	−0.21 [−0.68, 0.26]
Neubert 2023	2.28	19.66	67	3.53	17.7	72	6.3%	−0.07 [−0.40, 0.27]
Urech 2018	−8.59	17.78	65	0	17.78	64	6.2%	−0.48 [−0.83, −0.13]
**Subtotal (95% CI)**			**218**			**222**	**29.8%**	**−0.23 [−0.41, −0.04]**
Heterogeneity: $Tau^2 = 0.00$; $Chi^2 = 3.85$, df = 4 (P = 0.43); $I^2 = 0\%$								
Test for overall effect: Z = 2.34 (P = 0.02)								
**2.12.8 Autoimmune**								
Ferwerda 2017	−3.85	11.47502941	46	−2.36	10.40024519	59	6.2%	−0.14 [−0.52, 0.25]
Menting 2017	−19.4	10.04639239	60	−5.6	9.02662728	60	6.1%	−1.44 [−1.84, −1.03]
van Beugen 2016	−6.93	11.67224057	44	−0.73	9.79229799	49	6.1%	−0.57 [−0.99, −0.16]
Watt 2023	−2.7	16.20833119	43	0.8	15.91508718	44	6.1%	−0.22 [−0.64, 0.21]
**Subtotal (95% CI)**			**193**			**212**	**24.4%**	**−0.59 [−1.18, −0.00]**
Heterogeneity: $Tau^2 = 0.32$; $Chi^2 = 25.29$, df = 3 (P < 0.0001); $I^2 = 88\%$								
Test for overall effect: Z = 1.96 (P = 0.05)								
**2.12.9 Neurological**								
Fischer 2015	−4.03	14.94901669	35	−0.97	15.65690902	36	5.9%	−0.20 [−0.66, 0.27]
Friesen 2017	−4.61	14.10269123	30	−1.33	13.58562108	30	5.8%	−0.23 [−0.74, 0.27]
İşcan Ayyildiz 2024	−1.42	0.7332803	30	0.08	0.65023073	30	5.4%	−2.14 [−2.78, −1.49]
Moss-Morris 2012	−9	5.98862255	20	−1.96	4.61740187	16	5.2%	−1.27 [−2.00, −0.54]
Pöttgen 2018	−2.74	6.7	139	0	6.7	136	6.5%	−0.41 [−0.65, −0.17]
Schröder 2014	−0.63	0.45133136	24	0.37	0.39153544	32	5.3%	−2.36 [−3.06, −1.66]
Titcomb 2023	−0.68	0.21517435	46	0.04	0.24062419	49	5.5%	−3.12 [−3.73, −2.52]
Williams 2010	−0.8	11.3661779	59	−0.1	11.95784261	59	6.2%	−0.06 [−0.42, 0.30]
**Subtotal (95% CI)**			**383**			**388**	**45.8%**	**−1.19 [−1.90, −0.48]**
Heterogeneity: $Tau^2 = 0.98$; $Chi^2 = 130.21$, df = 7 (P < 0.00001); $I^2 = 95\%$								
Test for overall effect: Z = 3.29 (P = 0.001)								
**Total (95% CI)**			**794**			**822**	**100.0%**	**−0.74 [−1.09, −0.39]**
Heterogeneity: $Tau^2 = 0.48$; $Chi^2 = 174.92$, df = 16 (P < 0.00001); $I^2 = 91\%$								
Test for overall effect: Z = 4.16 (P < 0.0001)								
Test for subgroup differences: $Chi^2 = 7.55$, df = 2 (P = 0.02), $I^2 = 73.5\%$								

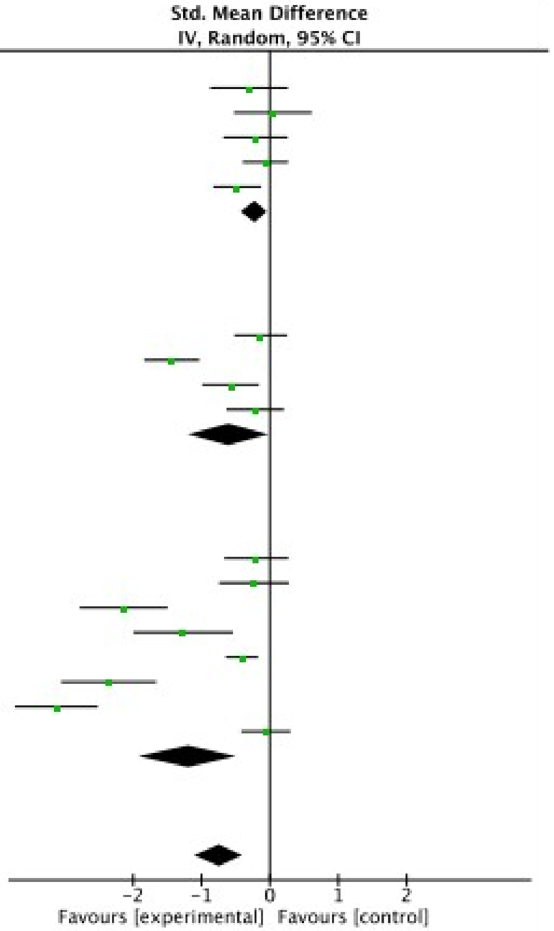

Supplement: Supplementary file 8 [file pdig.0000878.s005.docx]
